# ERP correlates of shared control mechanisms involved in saccade preparation and in covert attention

**DOI:** 10.1016/j.brainres.2006.12.007

**Published:** 2007-03-02

**Authors:** Martin Eimer, Jose Van Velzen, Elena Gherri, Clare Press

**Affiliations:** a Department of Psychology, Birkbeck College, University of London, Malet Street, London WC1E 7HX, England, UK; b Department of Psychology, Goldsmiths College, University of London, UK; c Department of Psychology, University of Modena and Reggio Emilia, Italy; d Department of Psychology, University College, University of London, UK

**Keywords:** Spatial attention, Eye movements, Attentional control, Vision, Event-related brain potentials

## Abstract

We investigated whether attention shifts and eye movement preparation are mediated by shared control mechanisms, as claimed by the premotor theory of attention. ERPs were recorded in three tasks where directional cues presented at the beginning of each trial instructed participants to direct their attention to the cued side without eye movements (Covert task), to prepare an eye movement in the cued direction without attention shifts (Saccade task) or both (Combined task). A peripheral visual Go/Nogo stimulus that was presented 800 ms after cue onset signalled whether responses had to be executed or withheld. Lateralised ERP components triggered during the cue–target interval, which are assumed to reflect preparatory control mechanisms that mediate attentional orienting, were very similar across tasks. They were also present in the Saccade task, which was designed to discourage any concomitant covert attention shifts. These results support the hypothesis that saccade preparation and attentional orienting are implemented by common control structures. There were however systematic differences in the impact of eye movement programming and covert attention on ERPs triggered in response to visual stimuli at cued versus uncued locations. It is concluded that, although the preparatory processes underlying saccade programming and covert attentional orienting may be based on common mechanisms, they nevertheless differ in their spatially specific effects on visual information processing.

## 1 Introduction

Our cognitive system has evolved to enable the adaptive control of behaviour in complex and constantly changing environments where the selective processing of relevant sensory input and the selection of appropriate motor outputs are continuously required. In fact, the capacity to select and process currently relevant sensory information (selective attention) and the ability to select, program and activate specific responses may be closely linked. According to the premotor theory of attention (Rizzolatti et al., 1994), the mechanisms responsible for the control of selective attention and the mechanisms underlying response selection and activation are implemented by common mechanisms. This theory assumes that goal-directed movements and shifts of spatial attention are mediated by shared control structures and that attention shifts are triggered whenever such structures are activated during response preparation. Thus, in order to initiate intentional (endogenous) shifts of spatial attention, specific response programs (such as plans for manual or saccadic eye movements) have to be activated. In the case of covert attentional orienting, response programming can take place without execution of the peripheral motor parts of these programs.

There is now substantial empirical evidence to support the claim of the premotor theory that the programming of saccadic eye movements and shifts of visual attention are closely linked. Behavioural experiments have demonstrated that attentional shifts towards saccade target locations are triggered during eye movement preparation (Hoffman and Subramaniam, 1995; Irwin and Gordon, 1998) and that these can affect performance even before the eyes have begun to move (Deubel and Schneider, 1996). The current focus of visual attention can affect eye movement trajectories (Sheliga et al., 1995), even when saccades are elicited via stimulation of the superior colliculus (Kustov and Robinson, 1996). Eye movement preparation can also produce spatially selective attentional effects on the processing of auditory (Rorden and Driver, 1999) and tactile stimuli (Rorden et al., 2002).

While such behavioural findings demonstrate the existence of strong links between attention and eye movement preparation, they can only provide indirect evidence for the core claim of the premotor theory that the control of attention and the programming of eye movements are mediated by common structures. In order to test this hypothesis directly, the control processes activated during saccade preparation and during covert shifts of spatial attention need to be measured and experimentally manipulated. Several recent studies have employed different neuroscientific and neuropsychological methods to achieve this objective. Functional imaging experiments have uncovered considerable overlap of dorsal frontoparietal control structures that are activated during covert shifts of visual attention and during saccade preparation (Corbetta et al., 1998; Nobre et al., 2000a; Perry and Zeki, 2000; Beauchamp et al., 2001). Patient studies have demonstrated that peripheral oculomotor deficits can impair spatial attention (Craighero et al., 2001; Smith et al., 2004). Transcranial magnetic stimulation (TMS) over the frontal eye fields was found to modulate attentionally guided performance in visual search tasks (Muggleton et al., 2003), and direct electrical stimulation of the frontal eye fields can improve monkeys' performance in spatial attention tasks (Moore and Fallah, 2001). Taken together, these findings provide strong evidence in support of the claim of the premotor theory that attentional orienting and saccade programming are closely linked and that they may be based on shared control mechanisms.

In spite of the evidence discussed above, the exact nature of such links between shifts of attention and eye movement preparation remains elusive. The aim of the present event-related brain potential (ERP) study was to independently measure and directly compare electrophysiological correlates of eye movement preparation and of covert attentional shifts. ERPs have already been successfully employed as on-line electrophysiological measures of control processes that are activated when shifts of attention are programmed and executed. Numerous studies have used attentional cueing procedures, where symbolic attentional precues such as left-pointing and right-pointing arrows presented at the start of each trial instruct participants to shift their attentional focus covertly (that is, without moving their eyes) towards a cued location in anticipation of task-relevant visual events. To identify ERP components sensitive to the direction of cued attentional shifts, ERP waveforms triggered in response to cues directing attention to the left side were compared to ERPs elicited during rightward attentional shifts (cf. Harter et al., 1989; Yamaguchi et al., 1994; Nobre et al., 2000b; Hopf and Mangun, 2000). An enhanced negativity at anterior recording sites contralateral to the cued side of an attentional shift (‘anterior directing attention negativity’, ADAN) was followed by a contralateral posterior positivity (‘late directing attention positivity’, LDAP). These lateralised ERP components were interpreted as reflecting successive phases in the control of visual–spatial attention, such as the initiation of an attention shift and the preparatory activation of visual brain areas. Similar components are elicited not only during shifts of visual attention, but also when participants direct their attention to the location of expected auditory or tactile events (Eimer et al., 2002, 2003; Eimer and Van Velzen, 2002; Van Velzen et al., 2002), suggesting that they might reflect the activity of a modality-unspecific attentional control system (see Eimer et al., 2002 for more details). It should be noted that, in addition to the ADAN and LDAP, a posterior contralateral negativity triggered within 250 ms after cue onset (‘early direction attention negativity’, EDAN) has been observed in several previous ERP studies of spatial orienting. However, this component appears to reflect the processing of non-symmetrical cue stimuli such as arrows (see Van Velzen and Eimer, 2003 for evidence) and is thus unlikely to be generated by processes that are directly involved in the control of anticipatory attentional shifts.

If the claim of the premotor theory that covert shifts of attention and response programming are mediated by shared control structures is correct, lateralised ERP components previously observed during attentional orienting should also be found under conditions in which participants are instructed to prepare manual responses or eye movements instead. Initial evidence for this prediction was found in a recent ERP experiment (Eimer et al., 2005) where participants had to prepare to lift their left or the right index finger (as indicated by a cue presented at the start of each trial). ADAN and LDAP components were elicited during unimanual response preparation and were very similar in terms of their amplitudes and latencies to the components triggered during covert attention shifts (see also Wauschkuhn et al., 1998; Verleger et al., 2000; Van der Lubbe et al., 2000 for previous investigations of lateralised ERP components elicited during response preparation). This finding was recently confirmed by Praamstra et al. (2005), who directly compared lateralised ERP components triggered during covert shifts of spatial attention and the preparation of unimanual responses. In an attention task, participants were cued to shift attention to the left versus right side. In a motor task, the cue instructed them to prepare a left versus right manual response. In an attention–motor task, the cue indicated both the direction of an attentional shift and the side of an upcoming manual response. Praamstra et al. (2005) not only found that ADAN and LDAP components were present in all three task conditions, but also that there was no difference in the amplitudes or scalp topographies of these components between tasks. Using dipole source analyses, they localised the generator processes responsible for the ADAN and LDAP in lateral premotor and occipital cortex, respectively.

Along similar lines, two recent ERP studies have investigated links between attention shifts and the preparation of saccadic eye movements. Van der Lubbe et al. (2006) compared ERP lateralisations triggered in the cue–target interval in response to cues indicating the direction of a covert shift of attention and in response to cues indicating the direction of an upcoming saccade. ADAN and LDAP components were observed in both tasks, suggesting that the neural systems involved in covert attentional orienting and in saccade preparation show considerable overlap. However, another recent experiment by Van der Stigchel et al. (2006) obtained partially conflicting results. In this study, where cues indicated the location of a subsequently presented saccade target on the left or right side, the ADAN component was again elicited during saccade preparation, but the LDAP was notably absent. Van der Stigchel et al. (2006) suggested that, while the ADAN may reflect the activation of neural structures involved in both covert attention and oculomotor preparation, the LDAP is not specifically involved in the control of eye movements.

A general and difficult problem for studies aiming to dissociate the contributions of attentional orienting and saccade preparation to lateralised ERP components is that the preparation of eye movements is likely to be accompanied by shifts of attention towards saccade targets on the left or right side. For example, Van der Lubbe et al. (2006, Exp.1) cued participants to make an eye movement towards a target circle that was filled with horizontal or vertical lines. Because this target could appear on the uncued side on 15% of all trials, covert attentional shifts towards the cued side were most likely triggered during the cue–target interval in order to facilitate the detection of the presence or absence of a saccade target on this side. Any lateralised ERP components elicited during saccade preparation might therefore exclusively reflect such attention shifts and could thus be entirely unrelated to eye movement preparation. Van der Lubbe et al. (2006) explicitly acknowledged this problem and therefore conducted a second experiment where participants were simply cued to make an eye movement towards one of two unfilled target circles on the left or right side about 1 s after cue onset. Because ADAN and LDAP components were triggered in this second experiment, even though no perceptual discrimination was required at the cued saccade target location, Van der Lubbe et al. (2006) concluded that these components reflect saccade programming in the absence of attentional orienting. However, one could argue that, even under these conditions, covert attentional shifts towards the continuously visible cued target circles may still have regularly occurred in the course of saccade preparation, given that such shifts may facilitate the encoding of saccade target locations. In order to conclusively dissociate saccade preparation from attentional orienting, experimental procedures are needed that explicitly discourage participants from moving their attention towards an anticipated saccade target location. The aim of the present ERP study was to employ such a procedure in order to find out whether lateralised ERP components (ADAN, LDAP) will still be elicited during saccade preparation in the absence of any concomitant covert attention shifts.

We compared ERP correlates of attention shifts and lateralised ERP modulations elicited during the preparation of leftward versus rightward saccadic eye movements, using procedures similar to those employed by Praamstra et al. (2005) in their investigation of links between spatial attention and manual response preparation. A trial-by-trial cueing procedure was used where visual precues at fixation were followed after 700 ms by unilateral peripheral visual target or non-target stimuli (red or green LED flashes) that were delivered with equal probability on the left or right side. Three task conditions were delivered in separate blocks, which differed with respect to task instructions and the response modality (eye movements versus vocal responses), but were otherwise identical in terms of the physical characteristics and spatial layout of the visual stimuli involved. The Covert task used standard endogenous attentional orienting procedures. Participants were instructed to maintain central fixation, to direct their attention to the side indicated by the cue and to respond vocally (by saying “yes”) whenever a visual Go stimulus was presented on the cued side. Responses had to be withheld to visual non-target stimuli on the cued side and to all stimuli on the uncued side. In this task, where cues informed participants about the location of upcoming task-relevant visual events, covert endogenous attention shifts should be triggered without intentional saccade preparation as eye movements were strictly discouraged. The Combined task was equivalent to the Covert task, except that vocal responses were now replaced by eye movements. Participants were instructed to prepare a saccade towards the LED on the side indicated by the cue and to execute this eye movement whenever a Go stimulus was presented on this cued side. Eye movements had to be withheld in response to non-target stimuli on the cued side and to all stimuli on the uncued side. In this task, where cues signalled the direction of an anticipated saccade as well as the location of an upcoming potentially response-relevant peripheral visual stimulus, saccade preparation and endogenous attention shifts should be elicited in parallel (analogous to Van der Lubbe et al., 2006, Exp.1). The critical Saccade task was equivalent to the Combined task, except that participants were now instructed to execute an eye movement towards the LED on the cued side whenever a visual target stimulus was detected on either side. Here, cues specified saccade direction, but were not informative with respect to the location of task-relevant visual events. Participants were told that target stimuli were equally likely to be delivered at the cued saccade target location or on the opposite side in order to discourage any endogenous attentional orienting towards the cued side. This Saccade task should thus provide ‘pure’ measures of ERP correlates of saccade preparation that are uncontaminated by any strategic covert attention shifts. On trials where target stimuli were delivered at the cued location, a prosaccade (a saccade towards a visual target) was required, while an antisaccade (a saccade away from a target) was required on trials where these stimuli were presented on the uncued side.

Lateralised ERP components triggered during the cue–target interval were directly compared between these three tasks to investigate the relative contributions of saccade programming and attentional orienting. For the Covert task, lateralised ERP components (ADAN, LDAP) analogous to those previously observed during cued shifts of endogenous spatial attention (cf. Harter et al., 1989; Yamaguchi et al., 1994; Nobre et al., 2000b; Hopf and Mangun, 2000; Eimer et al., 2002) were expected. The critical question was whether similar or different components would be found for the Saccade task. If the shared control processes activated during saccade programming and attentional orienting are identical, lateralised components elicited during the cue–target interval in this task should be very similar to components observed in the Covert task. In contrast, if eye movement control and the control of spatial attention are based on entirely distinct neural substrates, very different patterns of ERP effects should be found during side-specific preparation in the Saccade and the Covert tasks, with effects for the Combined task possibly reflecting the joint contributions of task-specific ERP modulations elicited during saccade preparation and covert orienting, respectively.

In addition to comparing ERP correlates of attentional orienting and saccade programming, another objective of the present experiment was to investigate whether preparatory attention and eye movement preparation have similar spatially specific effects on the processing of visual stimuli. There is substantial behavioural evidence that saccade preparation results in systematic modulations of visual processing (see above). Furthermore, numerous ERP studies have demonstrated that covert shifts of visual attention result in enhanced early visual P1 and N1 components for attended relative to unattended visual stimuli, as well as in later sustained attentional negativities (cf. Eason, 1981; Mangun and Hillyard, 1991; Eimer, 1994). Attentional modulations of sensory-specific P1 and N1 components are usually interpreted as evidence for the intraperceptual sensory gating of attended locations within visual perception (Mangun, 1995), while longer-latency effects are likely to reflect attentional modulations of post-perceptual processes (Mangun and Hillyard, 1991). Investigating whether similar effects can also be found as a consequence of saccade preparation can be seen as a critical test of the premotor theory of attention. If covert spatial orienting and eye movement preparation are based on shared neural control mechanisms, as claimed by this theory, they should result in a very similar pattern of spatially specific modulations of visual ERPs.

In the only previous ERP study to date that investigated the impact of saccade preparation on visual processing, Van der Stigchel et al. (2006) failed to find any modulations of visual P1 and N1 amplitudes. These authors suggested that this negative result may have been due to the minimal visual demands of their task, which required participants only to detect, but not to identify saccade targets. If this was the case, effects of saccade preparation on early visual components might be uncovered in the present experiment where a visual Go/Nogo stimulus discrimination was required on every trial. To test this prediction, we measured ERPs elicited in response to peripheral visual stimuli at cued versus uncued locations, separately for the Covert, Saccade and Combined tasks. In order to avoid contamination of visual ERPs by eye movements or vocal responses to target stimuli, these analyses were based exclusively on trials where non-target stimuli were presented, and no saccades or vocal responses were executed. For the Covert task, results were expected to confirm previous findings that visual–spatial attention results in modulations of sensory-specific P1 and N1 components, as well as in a subsequent negativity for visual stimuli at cued versus uncued locations beyond 200 ms post-stimulus. The critical new question was how spatial cueing would affect visual ERPs in the two other tasks. If attention shifts and saccade preparation are based on common underlying mechanisms, spatially selective modulations of visual ERPs should be very similar in all three tasks. In contrast, if saccade preparation and attentional covert orienting are mediated by anatomically and functionally distinct control processes, these processes might have different effects on the processing of subsequently presented visual events. This should be reflected by systematic differences in the pattern of spatial cueing effects on visual ERPs observed for the Saccade and Covert tasks, with cueing effects in the Combined task possibly reflecting the joint contribution of the effects observed in the two ‘pure’ tasks.

## 2 Results

### 2.1 Behavioural performance

Saccade RTs were faster in the Combined task than in the Saccade task (375 vs. 438 ms; *t*(17) = 5.6; *p* < .001). As expected, prosaccades were faster than antisaccades in the Saccade task (421 vs. 455 ms; *t*(17) = 2.4; *p* < .03). Vocal RTs in the Covert task (532 ms) were considerably slower than saccade latencies in the other two tasks (both *t*(17) > 5.4; both *p* < .001). RTs did not differ significantly as a function of target stimulus location or response direction (for eye movements).

False alarms occurred on 4.1% (Saccade task), 3.8% (Combined task), and 0.6% (Covert task) of all non-target trials. Participants failed to respond on 2.4% (Saccade task), 0.8% (Combined task), and 5.4% (Covert task) of all trials where relevant targets were presented. In the Saccade task, failures to respond to relevant targets were more frequent when targets required an antisaccade than when a prosaccade was required (3.3% vs. 1.5%; *t*(17) = 3.0; *p* < .01). In addition, incorrect saccades (i.e., saccades towards the uncued side) were observed on 3.2% of all trials in which an antisaccade was required, and only on 1% of all trials in which a prosaccade was required (*t*(17) = 3.2; *p* < .005).

### 2.2 ERPs in the cue–target interval: preparatory processes activated during attentional orienting and saccade programming

**Fig. 1:**
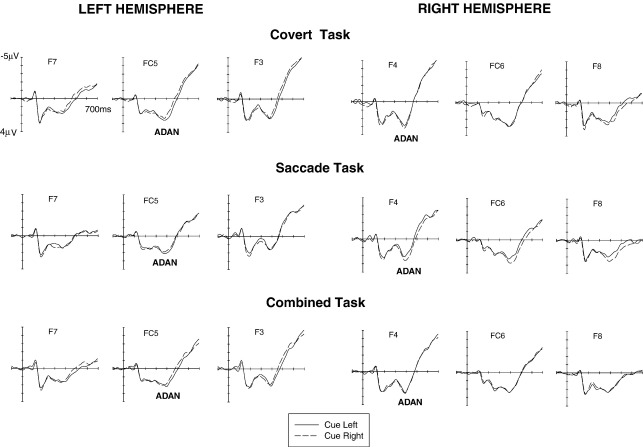
Grand-averaged ERPs elicited during the cue–target interval in the 700 ms interval following cue onset relative to a 100 ms precue baseline. These ERPs were obtained at lateral anterior electrodes in the Covert task (top panel), the Saccade task (middle panel), and the Combined task (bottom panel) in response to left cues (solid lines) and right cues (dashed lines). ADAN: anterior directing attention negativity.

**Fig. 2:**
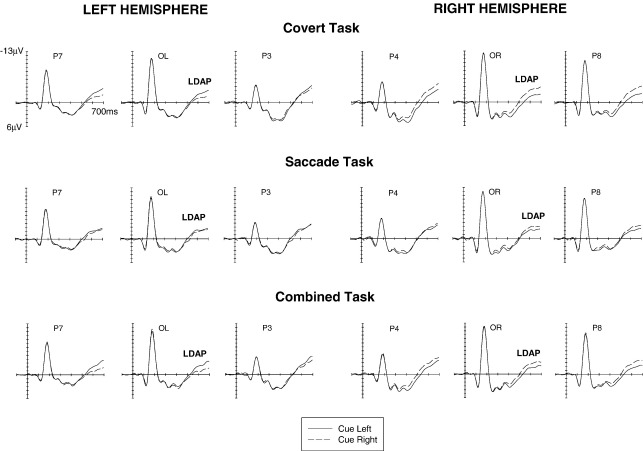
Grand-averaged ERPs elicited during the cue–target interval in the 700 ms interval following cue onset relative to a 100 ms precue baseline. These ERPs were obtained at lateral posterior electrodes in the Covert task (top panel), the Saccade task (middle panel) and the Combined task (bottom panel) in response to left cues (solid lines) and right cues (dashed lines). LDAP: late directing attention positivity.

Fig. 1 shows ERPs elicited in response to left and right cues in the interval between cue onset and the onset of the subsequent imperative visual stimulus at anterior electrode pairs over the left and right hemisphere in the Covert task (top panels), the Saccade task (middle panels) and the Combined task (bottom panels). Fig. 2 shows analogous ERPs for the cue–target interval at lateral posterior electrode pairs. These figures suggest that cue direction had systematic effects on ERPs elicited in the cue–target interval in all three tasks. Starting at about 350 ms after cue onset, ERPs were more negative at anterior electrodes contralateral as compared to ipsilateral to the side indicated by the cue, analogous to the anterior directing attention negativity (ADAN) observed in previous studies. This lateralised component appeared to be present for all three task conditions (see Fig. 1). Towards the end of the cue–target interval, ERPs were more positive at posterior electrodes contralateral to the cued side, in line with the late directing attention positivity (LDAP) reported in previous investigations. This LDAP component appeared to be smaller in the Saccade task than in the two other task conditions (see Fig. 2).

**Fig. 3:**
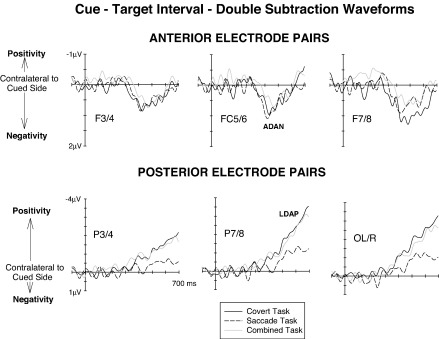
Difference waveforms obtained during the 700 ms cue–target interval at anterior (top panel) and posterior (bottom panel) lateral electrode pairs in the Covert task (black solid lines), the Saccade task (black dashed lines) and the Combined task (grey solid lines). Enhanced negativities contralateral to the cued side are reflected by positive values (downward deflections), and enhanced contralateral positivities are reflected by negative values (upward deflections).

The difference waves shown in Fig. 3 are included to further illustrate the amplitudes and the time course of these lateralised ERP modulations elicited during the cue–target interval in the three task conditions. These waveforms are shown solely to simplify graphical presentation, and not for statistical analysis. They were computed by subtracting ERPs in response to right cues from ERPs elicited by left cues and then subtracting the resulting difference waves for right-hemisphere electrodes from the difference waves for homologous electrodes over the left hemisphere. In the resulting double subtraction waveforms, an enhanced negativity at electrodes contralateral to the side indicated by the cue is reflected by positive amplitude values (downward-going deflections), while a contralateral positivity is indicated by negative values (upward-going deflections). Fig. 3 shows difference waveforms obtained for anterior (top), and posterior (bottom) electrode pairs, separately for the Covert task (black solid lines), the Saccade task (black dashed lines) and the Combined task (grey solid lines). While the anterior contralateral negativity (ADAN) appears to be similar in size for all three tasks, the amplitude of the contralateral positivity at posterior electrodes (LDAP) seems to be substantially attenuated for the Saccade task relative to the Covert and Combined tasks (see Fig. 3, bottom panels).

These informal observations were confirmed by statistical analyses. In the 350–500 ms interval, a significant hemisphere × cue direction interaction was present at lateral anterior electrodes (*F*(1,17) = 27.8; *p* < .001), reflecting the presence of the ADAN component as shown in Figs. 1 and 3. Importantly, there was no indication of any task condition × hemisphere × cue direction interaction (*F* < 1), suggesting that this ADAN was elicited in a comparable fashion for all three task conditions. The presence of an ADAN in all three tasks was confirmed in analyses conducted separately for each task condition, which revealed significant hemisphere × cue direction interactions at anterior electrode pairs for all three tasks (all *F*(1,17) > 4.4; all *p* < .05). At lateral central electrodes, a hemisphere × cue direction × electrode site interaction was present (*F*(2,34) = 5.3; *p* < .03; *ε* = .655) for the 350–500 ms interval. A hemisphere × cue direction interaction was significant at C3/4 only (*F*(1,17) = 7.0; *p* < .02), reflecting the presence of the ADAN at this electrode pair (not shown in Fig. 1).

At lateral posterior electrodes, a hemisphere × cue direction interaction was also found during the 350–500 ms time window (*F*(1,17) = 5.5; *p* < .04). To explore whether this was due to the relatively early onset of the posterior LDAP component in the present study (see Figs. 2 and 3), separate analyses were conducted for the first and second part of this time window (350–420 ms and 425–500 ms, respectively) at lateral posterior electrode pairs. As expected, a significant hemisphere × cue direction interaction was only present for the 425–500 ms interval (*F*(1,17) = 6.4; *p* < .03), reflecting the emergence of the LDAP component. A task condition × hemisphere × cue direction interaction (*F*(2,34) = 4.1; *p* < .05; *ε* = .691) was present during this interval, and follow-up analyses revealed significant hemisphere × cue direction interactions for the Covert and Combined tasks (both *F*(1,17) > 4.6; both *p* < .05). No such interaction was found during this time interval for the Saccade task (*F* < 1.6), indicating that LDAP onset was delayed in this task.

In the final 200 ms of the cue–target interval (500–700 ms after cue onset), the presence of the LDAP component was reflected by a significant hemisphere × cue direction interaction at lateral posterior electrode pairs (*F*(1,17) = 24.2; *p* < .001). Importantly, a significant three-way interaction was also obtained (task condition × hemisphere × cued direction: *F*(2,34) = 14.6; *p* < .001; *ε* = .918), suggesting that there were systematic differences in LDAP amplitudes between the three tasks, with larger LDAP components in the Covert and Combined tasks relative to the Saccade task (see Figs. 2 and 3). Analyses conducted separately for each task condition revealed hemisphere × cue direction interactions not just for the Covert task (*F*(1,17) = 48.0; *p* < .001) and the Combined task (*F*(1,17) = 18.6; *p* < .001), but also for the Saccade task (*F*(1,17) = 7.5; *p* < .02), thereby demonstrating that the LDAP component was reliably elicited in all three tasks. To investigate whether the component was attenuated for the Saccade task relative to the Covert task, the data for these two tasks were analysed together (with task condition now a two-level factor). A significant task condition × hemisphere × cue direction interaction was obtained (*F*(1,17) = 31.6; *p* < .001), demonstrating that the LDAP was smaller in the Saccade relative to the Covert task. When the data for the Saccade and Combined tasks were analysed together, the task condition × hemisphere × cue direction interaction was again significant (*F*(1,17) = 19.8; *p* < .001), indicating that the LDAP for the Saccade task was smaller than the LDAP triggered in the Combined task. In contrast, when the data from the Covert and Combined tasks were analysed together, no indication of any task condition × hemisphere × cue direction interaction was obtained (*F* < 1), thus confirming the impression suggested by Figs. 2 and 3 that LDAP amplitudes did not differ between these two tasks.

No hemisphere × cue direction interaction was present during the 500–700 ms time window at lateral anterior sites. At lateral central electrodes, a hemisphere × cue direction × electrode site interaction was present (*F*(2,34) = 11.2; *p* < .001; *ε* = .884) for the 500–700 ms interval. A hemisphere × cue direction interaction was found at CP5/6 only (*F*(1,17) = 10.8; *p* < .01), reflecting the presence of an LDAP (not shown in Fig. 2). Here, a task condition × hemisphere × cue direction interaction was also present (*F*(2,34) = 4.9; *p* < .02; *ε* = .930), again due to the attenuation of the LDAP in the Saccade task.

### 2.3 ERPs to peripheral visual non-targets: spatially specific effects of covert orienting and saccade programming on visual processing

**Fig. 4:**
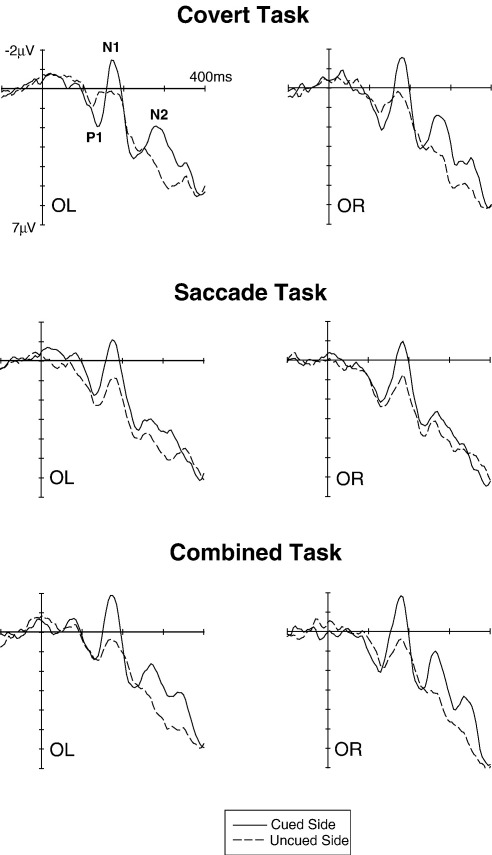
Grand-averaged ERPs elicited in response to visual non-target stimuli in the 400 ms interval following stimulus onset (relative to a 100 ms pre-stimulus baseline) at lateral occipital electrodes OL/OR. ERPs to non-target stimuli on the cued side (solid lines) or uncued side (dashed lines) are shown separately for the Covert task (top panel), the Saccade task (middle panel) and the Combined task (bottom panel).

**Fig. 5:**
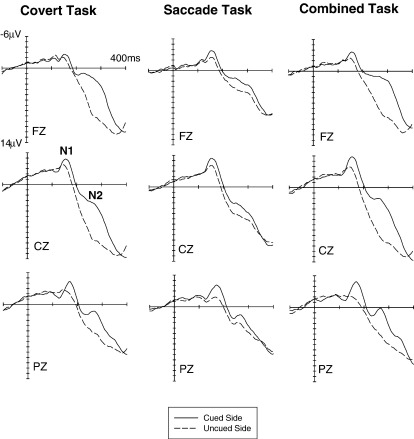
Grand-averaged ERPs elicited in response to visual non-target stimuli in the 400 ms interval following stimulus onset (relative to a 100 ms pre-stimulus baseline) at midline electrodes. ERPs to non-target stimuli on the cued side (solid lines) or uncued side (dashed lines) are shown separately for the Covert task (left panel), the Saccade task (middle panel) and the Combined task (right panel).

Fig. 4 shows ERPs triggered in response to visual non-target stimuli at cued locations (solid lines) and uncued locations (dashed lines) at lateral occipital electrodes OL/OR in the Covert task (top panel), the Saccade task (middle panel) and the Combined task (bottom panel). Fig. 5 shows visual ERPs elicited in these three task conditions at midline electrodes. While spatial cueing had systematic effects on visual ERPs in all three tasks, the size and direction of some of these effects differed between tasks. N1 amplitudes were consistently enhanced in response to visual stimuli at cued versus uncued locations in all three tasks. In contrast, effects of spatial cueing on P1 components appeared to go into opposite directions for the Covert task (larger P1 amplitudes for visual stimuli at cued locations) and the Saccade task (larger P1 amplitudes for uncued visual stimuli), with no clear P1 cueing effect in the Combined task. In addition, a sustained negativity for cued versus uncued visual stimuli was consistently present for all three tasks beyond 200 ms post-stimulus, although this effect appeared to be smaller in the Saccade task relative to the other two task conditions.

These observations were confirmed by statistical analyses. While there was no overall main effect of cue validity on P1 mean amplitudes (measured between 100 and 130 ms post-stimulus) at lateral posterior electrodes, a significant task condition × cue validity interaction (*F*(2,34) = 7.1; *p* < .01; *ε* = .892) was present. Follow-up analyses revealed a significant effect of cue validity on P1 amplitudes in the Covert task (*F*(1,17) = 7.5; *p* < .02), reflecting enhanced P1 components for visual stimuli on the cued (attended) relative to the uncued side (Fig. 4, top panel). In contrast, a main effect of cue validity in the Saccade task (*F*(1,17) = 5.3; *p* < .04) was due to the fact that ERP amplitudes in the P1 time range tended to be more negative in response to visual stimuli at cued versus uncued locations (Fig. 4, middle panel). No significant effect of cue validity on P1 amplitudes was present in the Combined task (*F*(1,17) = 2.5; *p* = .13).

In the N1 time range (160–200 ms), main effects of cue validity were present at lateral posterior, central, anterior as well as at midline sites (all *F*(1,17) > 17.8; all *p* < .001), demonstrating that N1 amplitudes were enhanced in response to visual stimuli on the cued versus uncued side (see Figs. 4 and 5). Importantly, and in contrast to the results obtained for the P1 component, there was no indication of any task condition × cue validity interactions at any recording site (all *F* < 1.7), suggesting that these N1 amplitude enhancements were triggered in an analogous fashion for all three tasks.

In the N2 time range (240–300 ms post-stimulus), a highly significant main effect of cue validity was obtained at midline electrodes (*F*(1,17) = 43.5; *p* < .001), due to the presence of enhanced negativities for visual stimuli presented on the cued versus uncued side (Fig. 5). A task condition × cue validity interaction (*F*(2,34) = 9.6; *p* < .002; *ε* = .702) suggested that the size of this effect differed between tasks, with smaller cueing effects in the Saccade task relative to the other two tasks. In analyses conducted separately for each task, main effects of cue validity were uniformly present (all *F*(1,17) > 8.6; all *p* < .01), confirming that enhanced negativities were triggered in the N2 time window by visual stimuli on the cued side in all three task conditions. However, when the ERP data for the Covert and Saccade tasks were analysed together (with task condition as two-level factor), a significant task condition × cue validity interaction was obtained (*F*(1,17) = 8.8; *p* < .01), demonstrating that these cueing effects were smaller for the Saccade task than for the Covert task. Similarly, when the data for the Combined and Saccade tasks were analysed together, a task condition × cue validity interaction (*F*(1,17) = 15.8; *p* < .001) indicated smaller cueing effects in the Saccade relative to the Combined task. In contrast, there was no task condition × cue validity interaction when the data from the Covert and Combined tasks were analysed together (*F* < 1), thus confirming that cueing effects in the N2 time range did not differ between these two tasks.

## 3 Discussion

To investigate the central claim made by the premotor theory of attention that attention shifts and saccade preparation are mediated by shared control mechanisms, we recorded ERPs under conditions in which central symbolic spatial cues indicated the direction of a covert endogenous attentional shift (Covert task), the direction of an upcoming eye movement (Saccade task) or both (Combined task). Responses were to be executed or withheld following a peripheral visual Go/Nogo stimulus that was presented 800 ms after cue onset. One set of analyses focussed on lateralised ERP components triggered during the cue–target interval, which are assumed to reflect preparatory attentional control mechanisms. Another set of analyses focussed on ERPs to subsequent visual non-target stimuli at cued versus uncued locations in order to gain insights into the nature of spatially selective modulations of visual processing induced by attentional orienting and saccade preparation, respectively.

In the Covert task, where participants directed their attention to the cued side in order to detect and vocally respond to visual target stimuli on this side, and no eye movements were allowed, systematic ERP lateralisations sensitive to the side of a cued attentional shift (ADAN, LDAP) were elicited during the cue–target interval. This confirms numerous earlier observations (cf. Harter et al., 1989; Yamaguchi et al., 1994; Nobre et al., 2000b; Hopf and Mangun, 2000; Eimer et al., 2002, 2003), although recent results (Green and McDonald, 2006) have suggested that the presence of an ADAN might sometimes depend on cue modality. ADAN and LDAP components have previously been interpreted as reflecting processes involved in the endogenous control of covert attentional orienting. If intentional shifts of attention are initiated via the activation of specific saccade programs, as claimed by the premotor theory, similar lateralised components should also be triggered under conditions in which cues signal the direction of an upcoming eye movement, even when there is no incentive for strategic covert attention shifts towards the cued side. The results observed in the Saccade task confirmed this prediction. In this task, where targets were equally likely to be presented on the cued and uncued side, and eye movement preparation should thus proceed in the absence of any concomitant strategic shifts of covert spatial attention, ADAN and LDAP components were clearly present. This result is in line with earlier observations by Van der Lubbe et al. (2006) and also confirms recent findings by Eimer et al. (2006), who directly compared ERP lateralisations during cued manual response preparation and saccade programming and showed that ADAN and LDAP components were elicited in both tasks. They are however partially inconsistent with the findings of Van der Stigchel et al. (2006) who failed to observe an LDAP during eye movement preparation. It is important to note that, for all of these previous studies, the possibility cannot be ruled out that covert shifts of attention were triggered in parallel with saccade preparation and that ADAN and LDAP components thus primarily or exclusively reflected such covert attentional orienting processes. However, the fact that these components were also reliably triggered in the Saccade task of the present study provides strong evidence against this interpretation.

The current results are therefore inconsistent with the view that saccade programming and spatial attention are controlled by anatomically and functionally independent mechanisms. If this was the case, qualitatively different patterns of lateralised ERP effects should have been observed in the cue–target interval for the Covert and Saccade tasks. The presence of ADAN and LDAP components in both these two ‘pure’ tasks suggests that there is considerable overlap in the neural mechanisms underpinning shifts of attention and eye movement preparation, as postulated by the premotor theory of attention.

It is also notable that ADAN and LDAP components did not differ between the Covert and Combined tasks in terms of their amplitudes and onset latencies. In the Combined task, participants prepared an eye movement towards the cued side and also shifted attention to this side in anticipation of task-relevant visual events. In the Covert task, cues also triggered endogenous attentional orienting, but eye movements were not allowed. The finding that equivalent lateralised components were elicited in these two tasks is exactly what would be predicted if endogenous covert attention shifts and eye movement preparation are functionally linked, as postulated by the premotor theory. If attention shifts are initiated by activating specific saccade programs, instructing participants to prepare eye movements in addition to shifting attention towards the cue location would be entirely redundant as such eye movements would have to be programmed in any case as a consequence of having to perform a covert attention shift. Analogously, if attentional orienting was required for saccade preparation, no differences between ADAN and LDAP components should be observed between the Covert and Combined tasks.

While the anterior ADAN component was triggered in a very similar fashion in all three tasks, the posterior LDAP was delayed and attenuated in the ‘pure’ Saccade task relative to the other two task conditions (see Figs. 2 and 3). This attenuation of the LDAP in the Saccade task might be related to the fact that, in contrast to the Combined task (where only prosaccades were required), prosaccades and antisaccades were equally likely in this task. The inclusion of antisaccade trials was an inevitable consequence of the necessity to use cues that were uninformative with respect to the side of task-relevant visual events in the Saccade task and thus to prevent strategic attention shifts towards expected locations of such events. However, including antisaccades might have increased participants' anticipation of spatial conflict while preparing cued eye movements, thereby possibly affecting spatially selective control processes that are reflected by the LDAP component. This could be further explored in studies where the likelihood of prosaccades and antisaccades is systematically manipulated. Alternatively, the attenuation of the LDAP in the Saccade task could more generally indicate that the neural processes responsible for the LDAP are more directly linked to covert attentional orienting than to saccade preparation. According to this interpretation, the neural basis of attention and saccade preparation would not be identical, but only partially overlapping. This possibility is supported by recent observations by Van der Lubbe et al. (2006, Exp.2), who also found that the LDAP was smaller during saccade programming than during covert attentional orienting.

Overall, the presence of similar lateralised ERP components during covert attentional orienting and saccade programming provides strong evidence for the claims of the premotor theory that the underlying neural mechanisms are closely linked. If covert attention and saccade preparation were based on non-overlapping neural networks, entirely different ERP components should have been obtained during these two types of preparatory processes. However, the fact that the LDAP was attenuated and delayed in the Saccade task relative to the other two tasks that involved covert attentional orienting also indicates that there may also be systematic differences in the neural mechanisms underlying saccade preparation and attentional orienting.

The analysis of ERPs elicited by peripheral visual stimuli at cued versus uncued locations revealed some important differences in the impact of covert attention and eye movement preparation on visual processing. The results obtained in the Covert task confirmed numerous previous findings (cf. Eason, 1981; Mangun and Hillyard, 1991; Eimer, 1994) that covert shifts of visual attention result in modulations of P1 and N1 components, as well as in a sustained attentional negativity at longer latencies (see Figs. 4 and 5). The important question addressed in the present study was whether analogous modulations of visual ERPs would also be triggered as a consequence of eye movement preparation, as would be expected if covert attentional orienting and saccade programming were based on shared mechanisms.

The results obtained in the Saccade task do not provide unequivocal support for the premotor theory of attention. Very similar enhancements of N1 amplitudes in response to visual stimuli at cued versus uncued locations were observed for all three task conditions. The fact that eye movement preparation in the Saccade task resulted in enhanced N1 amplitudes to visual stimuli at saccade target locations differs from previous findings by Van der Stigchel et al. (2006) who failed to obtain any effect of saccade preparation on P1 and N1 components. This notable difference might be related to differences in the visual processing demands between these two studies. Whereas a Go/Nogo stimulus discrimination was required in the present experiment, the task used by Van der Stigchel et al. (2006) only required participants to detect the onset of a saccade target. However, the impact of covert attention and saccade programming on the posterior P1 component was very different in the present experiment (see Fig. 4). While the expected pattern of enhanced P1 amplitudes to visual stimuli on the cued side was obtained in the Covert task, the opposite effect (larger P1 amplitudes for visual stimuli on the uncued side) was observed in the Saccade task. The Saccade task also differed from the Covert task with respect to the sustained attentional negativity triggered in the N2 time range. Although reliably present, its amplitude was significantly reduced (see Fig. 5). If attentional orienting and saccade preparation are based on shared neural substrates, and identical preparatory control processes are activated in both cases, these should have produced similar spatially selective modulations of visual ERPs. The differences in the cueing effects observed in the Covert task and in the Saccade task, and in particular the finding that P1 modulations of opposite polarity were elicited in these two tasks, suggest that there are systematic differences in the impact of eye movement preparation and covert shifts of attention on visual processing.

The reversed cueing effect on P1 amplitudes observed in the Saccade task could be linked to an active inhibition of saccade execution during the cue–target interval (see Van der Lubbe et al., 2006 for further discussion of saccade inhibition), which could have persisted beyond target onset and thus have affected early visual target processing in a spatially specific fashion. Alternatively, it is possible that saccade preparation results in a longer-latency sustained negativity that happens to overlap with the P1 component and specifically affects ERPs in response to visual stimuli at cued saccade target locations (see Van der Stigchel et al., 2006 for evidence for such longer-latency effects of saccade preparation). In this context, it is interesting to note that, in contrast to the Covert task, where normal P1 cueing effects were observed, there was no P1 modulation at all in the Combined task (see Fig. 4, bottom). This is remarkable given that cues were spatially predictive with respect to the location of task-relevant visual events in this task. The absence of any P1 modulation in the Combined task might be explained by assuming that cueing effects of opposite polarity are triggered in the P1 time range during covert attentional orienting and eye movement preparation and that these effects cancelled each other out in the Combined task where both covert attentional orienting and saccade programming were activated simultaneously. Further systematic ERP investigations of saccade programming are required to uncover the typical ERP signature of saccade preparation effects on visual ERPs and to explore the neural basis of these effects.

In summary, the present ERP study has provided new insights into the control processes underlying covert shifts of attention and eye movement preparation. We demonstrated that lateralised ERP components elicited in the cue–target interval are elicited regardless of whether participants are cued to endogenously shift their attention, to prepare an eye movement or both. This is in line with the claim of the premotor theory of attention that saccade preparation and attentional orienting are mediated by shared control structures. However, the presence of differential spatially specific modulations of early visual ERP components in the three tasks suggests that, in spite of the overlap between underlying control mechanisms, saccade preparation and covert shifts of attention differ in their impact on visual information processing.

## 4 Experimental procedures

### 4.1 Participants

Twenty paid volunteers participated in the experiment. One participant had to be excluded because of poor eye fixation control in the cue–target interval, and one further participant was excluded because of a large number of eye blinks. Thus 18 participants (11 females), aged 20–36 years (mean 26 years), remained in the sample. Sixteen participants were right-handed, two were left-handed and all had normal or corrected vision by self-report.

### 4.2 Stimuli and apparatus

Participants sat in a dimly lit experimental chamber, with a head-mounted microphone positioned 2 cm in front of the mouth, facing a computer monitor at a viewing distance of 57 cm. Cues consisted of two adjacent triangles, covered a total visual angle of 3.5° × 2.5° and were presented at the centre of the computer screen at an angle of about 30° below eye level. One of the triangles was red, the other was blue, and they always pointed in opposite directions (‘><’ or ‘<>’). The four possible combinations of cue colour and cue arrangement were equiprobable and randomly distributed in each block. A central fixation cross, located in the space between the two triangles, was continuously present throughout the experimental blocks. Peripheral visual stimuli were presented via LED ensembles, which consisted of six LED segments arranged in a circle plus one central segment. The angular size of each LED was 0.65°, the diameter of the circle was 2.4°. To specifically mark saccade target locations, LED circles were surrounded with white rings. Two LED ensembles were mounted to poles at an eccentricity of 39° to the left or right of fixation, at the same elevation as the central cues and fixation. All peripheral visual stimuli were presented for 100 ms, but differed in colour (green: non-targets; red: targets). The luminance of the green and red LED ensembles, measured with a SpectraScan PR650 luminance meter (Micron Techniques Ltd.) at a distance of 57 cm from the screen at eye level height, was 71.3 cd/m^2^ and 32.3 cd/m^2^, respectively.

### 4.3 Procedure

The experiment consisted of 18 experimental blocks with 80 trials per block. Each trial started with the presentation of a central cue (100 ms duration), which was followed after an interval of 600 ms by a visual peripheral stimulus (100 ms duration). Intertrial interval was 2000 ms. Three task conditions (Covert task, Saccade task and Combined task) were delivered, each consisting of six successive blocks. The order in which these tasks were delivered was balanced across participants. The Saccade and Combined tasks required eye movements in response to target stimuli, whereas the Covert task required vocal responses instead. In the Covert task, participants had to respond vocally (by saying “yes”) whenever a visual target (a red LED flash) was presented at the side indicated by the central cue on that trial. Visual non-targets (green LED flashes) on the cued side, as well as all visual stimuli on the uncued side were to be ignored, and central fixation had to be maintained. In the Combined task, participants had to execute a saccade towards the LED on the side indicated by the cue whenever a visual target stimulus (a red LED flash) was presented on this side, but to maintain central fixation when visual non-targets were presented on the cued side, and when visual stimuli were presented on the uncued side. In the Saccade task, participants had to execute an eye movement towards the LED on the side that was indicated by the cue whenever a red visual target was presented on either side and to withhold eye movements on trials where a green non-target was presented. Thus, the Saccade task differed from the Combined task in that the former required only prosaccades (towards the visual target location), whereas prosaccades and antisaccades (towards the LED on the side contralateral to the visual target) were equally likely in the Saccade task. In all three tasks, the relevant side (left or right) was indicated at the beginning of each trial by the direction of either the red or the blue central triangle. For half of the participants, blue triangles indicated the relevant side, while red triangles indicated the relevant side for the other half. Relevant left-pointing and right-pointing triangles were presented with equal probability to the left or right of fixation.

Each covert and combined block contained 48 trials where green visual non-targets were presented, with twelve trials per block for each combination of cue direction (left versus right) and visual stimulus side (left versus right). Red targets were presented in the remaining 32 trials per block. Twenty-four of these targets were delivered on the cued side (twelve left, twelve right) and thus required a vocal response or an eye movement. On eight trials per block, visual targets appeared on the uncued side (four left, four right), and no response was required on these trials. In order to keep the number of response trials as well as the number of trials per block identical across tasks, Saccade task blocks only contained 24 trials where a red visual target was presented. Six trials per block were delivered for each combination of cued side and visual stimulus side, and eye movements were required on all of these trials. In the remaining 56 trials of each Saccade block, green non-targets were presented with equal probability on the left or right side and following a left or right cue.

Task instructions were shown on the computer screen prior to the start of each block. Participants were instructed to use the information provided by the cue to direct their attention to the cued location (in the Covert task), to prepare an eye movement in the cued direction (in the Saccade task) or both (in the Combined task) in order to respond as quickly and accurately as possible to relevant visual targets, while withholding responses to all other stimuli. They were explicitly encouraged to maintain central eye fixation in the cue–target interval. Several training blocks were run prior to the beginning of each task condition. Eye movements were closely monitored during these training blocks. Whenever the horizontal EOG revealed that participants did not maintain central eye fixation, additional training blocks were run until fixation was regarded as satisfactory.

### 4.4 Recording and data analysis

EEG was recorded with Ag–AgCl electrodes and linked-earlobe reference from F7, F3, Fz, F4, F8, FC5, FC6, T7, C3, Cz, C4, T8, CP5, CP6, P7, P3, Pz, P4 and P8 (according to the 10–20 system) and from OL and OR (located halfway between O1 and P7, and O2 and P8, respectively). Horizontal EOG (HEOG) was recorded bipolarly from the outer canthi of both eyes. Electrode impedance was kept below 5 kΩ, and the impedances of the earlobe electrodes were kept as equal as possible. Amplifier bandpass was 0.1 to 40 Hz. EEG and EOG were sampled with a digitisation rate of 200 Hz and stored on disk. No additional filters were applied after recording.

EEG and EOG were epoched off-line into 1300 ms periods, starting 100 ms prior to cue onset and ending 600 ms after the onset of the peripheral stimulus. Separate averages were computed for ERPs recorded in the cue–target interval (relative to a 100 ms baseline preceding cue onset) and for ERPs elicited by subsequent visual peripheral stimuli (relative to a 100 ms baseline preceding the onset of these stimuli). ERPs in response to peripheral visual stimuli were computed for non-target trials only to avoid contamination by vocal responses or eye movements. Trials with vocal or eye movement responses to non-targets were excluded from EEG analysis, as were non-target trials with eyeblinks (Fpz exceeding ± 60 μV), small horizontal eye movements (HEOG exceeding ± 30 μV) or other artifacts (a voltage exceeding ± 80 μV at any electrode) in the interval between cue onset and 600 ms after peripheral visual stimulus onset. On average, 20% of all trials were excluded to the presence of artifacts, and trial exclusion rate did not exceed 35% for any participant. Averaged HEOG waveforms obtained for each participant and task condition in the cue–target interval in response to left versus right cues were scored for systematic deviations of eye position, which indicate residual tendencies to move the eyes towards the cued location. A residual HEOG deviation exceeding ± 3 μV led to the disqualification of one participant.

The EEG obtained in the cue–target interval was averaged for all combinations of task condition (Covert vs. Saccade vs. Combined task) and cue direction (left vs. right). Mean amplitude values were computed at lateral anterior sites (F7/8, F3/4, FC5/6), lateral central sites (T7/8, C3/4, CP5/6) and lateral posterior sites (P7/8, P3/4, OL/R) for successive pre-defined latency windows (350–500 ms and 500–700 ms after cue onset), which were identical to the analysis windows used in our previous studies (e.g., Eimer et al., 2002). Separate repeated-measures analyses of variance (ANOVAs) were conducted for lateral anterior, central and posterior sites. These analyses included the factors electrode site (F7/8 vs. F3/4 vs. FC5/6, for the anterior analysis, C3/4 vs. T7/8 vs. CP5/6, for the central analysis, and OL/R vs. P3/4 vs. P7/8, for the posterior analysis), task condition, cue direction and hemisphere (left vs. right). It is important to note that, in these analyses, the presence of ERP lateralisations sensitive to the side of a cued attention shift and/or the side of a cued eye movement is reflected by significant hemisphere × cued direction interactions.

The EEG obtained in response to peripheral visual non-target stimuli was averaged separately for all combinations of task condition, cue direction and stimulus side (left vs. right). Mean amplitude values computed for latency windows centred on the peak amplitudes of visual P1, N1 and N2 components (P1: 100–130 ms post-stimulus; N1: 160–200 ms post-stimulus; N2: 240–300 ms post-stimulus). Repeated-measures ANOVAs included the factors electrode site (F7/8 vs. F3/4 vs. FC5/6, for anterior electrodes, C3/4 vs. T7/8 vs. CP5/6, for central electrodes, OL/R vs. P3/4 vs. P7/8, for posterior electrodes, Fz vs. Cz. vs. Pz, for midline electrodes), hemisphere (left vs. right, for lateral electrodes only), cue validity (visual stimulus presented on cued vs. uncued side) and stimulus side. For brevity, we only report analyses of P1 amplitudes at lateral posterior sites and analyses of effects in the N2 time range for midline electrodes. For all analyses, Greenhouse–Geisser adjustments to the degrees of freedom were applied where appropriate.

Voice onset times in the Covert task were measured with a voice key. Saccade onset latencies in the Saccade and Combined tasks were measured on the basis of HEOG waveforms recorded after the onset of a peripheral visual stimulus. Saccade onset was defined as the latency (in ms post-stimulus) of the first data point within this interval exceeding a threshold of ± 80 μV (relative to a 100 ms pre-stimulus baseline), with saccade direction (left vs. right) indicated by the polarity of this value. Vocal and saccade response times (RTs) obtained on trials where participants responded correctly to relevant visual target stimuli were compared between task conditions with paired *t*-tests.
